# Introducing the PRIDAL model for linking routine health and identifiable patient reported questionnaire data

**DOI:** 10.23889/ijpds.v1i1.304

**Published:** 2017-04-19

**Authors:** Mark Kingston, Martin Heaven, Helen Snooks, Hayley Hutchings

**Affiliations:** Swansea University

## Objectives

- To present an efficient privacy-protecting model (PRIDAL) for linking routine and identifiable patient-reported research data
- To describe the application of the PRIDAL model with within a major portfolio trial linking a range of health and demographic datasets pertaining to >230,000 patients, alongside >2,300 questionnaires
- To summarise the lessons learned from the application of the PRIDAL model

## Approach

The rise in availability, quality and use of routine health data has resulted in well-developed methods for anonymised linkage of data from multiple sources. However methods for combining identifiable data (e.g. patient-reported questionnaires) with routine anonymised data are not yet tried and tested. Linking these data presents opportunities to improve the feasibility and effectiveness of observational and experimental studies, but emerging data linkage processes must address the appropriate balance between data security and usability. The Process for Routine and Identifiable Data Linkage (PRIDAL) was devised to efficiently link routine hospital data and patient-reported quality of life and quality of care questionnaire data as part of the PRISMATIC trial (http://www.trialsjournal.com/content/14/1/301). This is a mixed methods progressive cluster randomised trial of the efficacy of an emergency admission risk prediction tool in primary care, funded by the NIHR HS&DR programme.

PRIDAL was conceived by a group of specialists in e-trials, health informatics, information governance and process mapping who reviewed data sources, flows, owners, and security to develop a practical and intuitive process model. 

## Results

We will present the PRIDAL process model and describe its application in relation to the PRISMATIC study. We will demonstrate that the model achieves high data matching rates (>99%), and consider the lessons learnt from its application.

## Conclusion

The linking of routine health and patient self-reported data presents a valuable opportunity in health research, but clear, replicable models, are needed to support ethical and practical data linkage. We present the fully tested PRIDAL model as a potential solution.

